# Awareness of environmental carcinogens and cancer risk among Jordanians

**DOI:** 10.1186/s42506-024-00173-9

**Published:** 2024-11-04

**Authors:** Walaa B. El Gazzar, Qusai I. Al-Hashaikeh, Bara’ A. Al Maslooki, Doa K. Qarout, Youssef M. Abdin, Mohammad O. Hamad, Qutaiba A. Al Shuraiqi, Balqees F. Al-Madi, Joumana A. Bassiouni, Nashwa Nabil

**Affiliations:** 1 Department of Anatomy, Physiology and Biochemistry, Faculty of Medicine, The Hashemite University, Zarqa, Jordan; 2 Department of Medical Biochemistry and Molecular Biology, Faculty of Medicine, Benha University, Benha, Egypt; 3 Faculty of Medicine, The Hashemite University, Zarqa, Jordan; 4 American Digital Schools, Amman, 11941 Jordan; 5 Department of Community, Environmental and Occupational Medicine, Faculty of Medicine, Benha University, Benha, Egypt

**Keywords:** Environmental carcinogens, Cancer awareness, Cross-sectional descriptive survey, Risk factors

## Abstract

**Background:**

In light of the existing body of scientific data, many substances are now recognized or reasonably assumed to be human carcinogens. Public knowledge about modifiable environmental carcinogens is regarded as a crucial first step in primary prevention. This study aimed to assess Jordanians' awareness of some of the recognized environmental human carcinogens and general cancer information.

**Methods:**

This study was conducted using a cross-sectional descriptive survey based on a questionnaire completed by Jordanian participants aged 18 or above. The questions consisted of the following sections: socio-demographic characteristics, questions about the prevalent and non-prevalent cancer types in Jordan and general causes of cancer, closed-ended questions to evaluate knowledge about environmental carcinogens as well as factors that influence the development of cancer, source of knowledge about carcinogens and interest in learning about human carcinogens, and the best way for prevention of cancer.

**Results:**

A total of 579 questionnaires were completed. Among respondents, 55.6% (*n* = 322) had a knowledge score ≥ 8 indicating good knowledge. However, low awareness was demonstrated regarding cancer-causing substances such as wood dust, Nitrosamines, Aflatoxins, Formaldehyde, Naphthalene, Asbestos, Benzene, and Arsenic. A significant portion of the participants failed to categorize infectious pathogens linked to cancer as variables that either cause cancer or raise the risk of developing cancer.

**Conclusion:**

This study demonstrated a good level of awareness regarding some environmental carcinogens but also highlighted the lack of knowledge about other environmental carcinogens. These findings may provide a guide for future awareness programs by health authorities.

## Introduction

Cancer is among the leading causes of death and a major impediment to raising life expectancy worldwide. The burden of its incidence and mortality is expanding quickly on a global scale. The anticipated worldwide cancer burden is expected to reach 28.4 million cases in 2040, representing a 47% increase from 2020 [1].

Cancer is caused by mutations in specific genes that change the cell behaviour. Some of these genetic alterations are naturally brought about whenever a cell divides and DNA is replicated. However, there are also others that result from exposure to environmental factors that damage the DNA [2]. A carcinogen is any substance that can cause cancer. Based on the existing scientific knowledge, two organizations – the National Toxicology Program (NTP) and the International Agency for Research on Cancer (IARC) – have created lists of compounds that are either recognized as human carcinogens or reasonably anticipated to be so [3]. The NTP produces the Report on Carcinogens (RoC). This scientific and public health document lists and describes agents, substances, mixtures, or exposure conditions that may pose a cancer hazard to humans [4]. Among these carcinogens are cancer-causing substances such as aflatoxins, arsenic, and asbestos; infectious agents such as the Epstein-Barr Virus (EBV), Hepatitis B Virus & Hepatitis C Virus (HBV and HCV), Human Immunodeficiency Virus (HIV), and Human Papillomaviruses (HPVs); radiation, sunlight, and tobacco [2, 5].

Primary prevention is a major rationale for existing regulatory policies that seek to reduce the exposure of humans to substances that cause cancer. By limiting exposure, these policies enhance public health by lowering the number of cancer cases and reducing the financial burden associated with cancer. In addition, individuals can reduce their exposure to recognized carcinogens by giving up smoking, minimizing sun exposure, consuming less alcohol, and for those who are at the appropriate age, having HPV and HBV vaccination [4, 6]. Therefore, raising awareness among the general population about these modifiable environmental evidence-based risk factors is considered an important step in primary prevention. This study aimed to assess the Jordanians' general cancer knowledge and their awareness of some of the known environmental human carcinogens identified by The NTP in the NTP's 15th Report on Carcinogens.

## Methods

### Study design and population

This study was conducted using a cross-sectional descriptive survey based on an online questionnaire that was completed by the general Jordanian population where the inclusion criteria were: age 18 or above. The questions' content was developed based on the study's objectives and the body of research on environmental carcinogen awareness, which includes quantitative studies that used surveys and questionnaires to gather data from sizable participant cohorts [7, 8].

The questionnaire consisted of the following sections:

a Participants' characteristics: age, sex, residence, income, marital status, occupation, and family history of cancer.
b Questions about the prevalent and non-prevalent cancer types in Jordan, according to the GLOBOCAN estimates for 2020 in Jordan [9], and general causes of cancer.
c Questions assessing the general knowledge about environmental carcinogens and cancer risk including 19 closed-ended questions to evaluate knowledge about environmental carcinogens cited by The National Toxicology Programme (NTP) as known human carcinogens in the 15th Report on Carcinogens [4], such as ionizing radiation, UVR, smoking, second-hand smoking, alcohol, aflatoxins, arsenic, asbestos, benzene, naphthalene, coke oven emissions, formaldehyde, wood dust, nitrosamine, EBV, hepatitis B&C viruses, HIV, HPV, and Helicobacter pylori. One point was given for each correct answer and the total score of correct answers was calculated. Knowledge scores ranged from 1 to 19. Good knowledge of environmental carcinogens was defined as a total knowledge score ≥ 8 based on the median knowledge score. This section included also questions about the factors that affect a person's likelihood of developing cancer after being exposed to a carcinogen.
d The source of knowledge about carcinogens, interest in learning about human carcinogens, and the best way for prevention of cancer.

The content validity of the questionnaire was evaluated by 3 experts. Reliability of the risk factors knowledge scale was assessed using Cronbach’s alpha, which was calculated for all the items on the knowledge scale and was found to be equal to 0.907 indicating excellent reliability [10]. The questionnaire was translated to Arabic using forward and backward translation and then pilot-tested with a sample of 50 participants for understandability and consistency. The electronic questionnaire form was shared through social media platforms. Moreover, university students and employees, and employees in different governmental institutions and organizations were invited via email to participate in the anonymous online survey on Google Forms. Data were collected over the period from January to May 2023.

### Sample size

The sample size was calculated using Epi info Soft calculator version 3 according to the following equation:

$$\text{SS}={\text{Z}}^{2}*(\text{P})*(1-\text{P})/{\text{E}}^{2}$$

- Z = Z value (e.g. 1.96 for 95% confidence level)
- P = Prevalence of the condition
- E = Standard error expressed as decimal (e.g., 0.05).

The study power was 80% with a confidence level of 95%, and the anticipated percentage of public awareness of genetic and environmental carcinogens was estimated to be 66.6% [7]. The minimal calculated sample size was 342 subjects and a total of 579 online survey forms were received and determined to be complete and acceptable for analysis. This study was approved by the Institutional Review Board of the Hashemite University (approval No. 14/3/2022/2023).

### Knowledge scoring system

The environmental carcinogens knowledge score ranged from 1 to 19 (correct [true] answer = 1 score, wrong [false] answer = 0 score, not sure = 0 score). Scores < 50% ( < 8) were considered poor knowledge, and those ≥ 50% (≥ 8) were considered good knowledge [11, 12].

### Statistical analysis and data management

IBM-SPSS 25.0 (IBM-SPSS Inc., Chicago, IL, USA) was used for data analysis. To ascertain whether the distribution of the quantitative data was normal, the Kolmogorov–Smirnov test was employed. Frequency and percentage were used to summarise qualitative data, while the median (IQR) was utilized for quantitative data. Quantitative data were compared using the Mann–Whitney and Kruskall-Wallis tests. Multivariate logistic regression analysis was performed. To evaluate reliability, the Cronbach's alpha test was employed. All tests were two-sided. In this study, p < 0.05 was considered the acceptable threshold of significance, and p ≤ 0.01 was deemed highly statistically significant.

## Results

### Socio-demographic characteristics of the study sample

A total of 579 participants completed surveys. The participants' ages ranged from 18 to over 47 and the highest group was aged 18–27 years (38.4%, *n* = 222), Most of the study participants were females (69.4%, *n* = 402) and 48.9% (283) were married. Among all the research subjects, 81.5% held a bachelor’s degree or higher. The highest proportion of the study sample (61.3%) lived in Amman, 73.4% were employed and only 23.8% worked in the medical field. Regarding financial status, 50% of the participants earned between 500 and 1000 JD monthly, 28.8% had a family income of less than 500 JD monthly and only 21.2% had a family income of more than 1000 JD monthly. Of the participants, 35.1% (*n* = 203) had a family member who had been diagnosed with cancer (Table 1).

**Table 1:** Socio-demographic characteristics of the study sample, Jordan, 2023

Variables (*N* = 579)	Frequency (n)	Percentage (%)
**Gender**		
Male	177	30.6
Female	402	69.4
**Age**		
18–27	222	38.4
28–37	156	26.9
38–47	101	17.4
> 47	100	17.3
**Marital status**		
Single	276	47.7
Married	283	48.9
Divorced	10	1.7
Widowed	10	1.7
**Education level**		
Below University	107	18.5
University and Above	472	81.5
**Region of residence**		
Amman	355	61.3
Zarqa	109	18.8
Irbid	15	2.6
Mafraq	20	3.5
Karak	4	0.7
Ajloun	2	0.3
Aqaba	3	0.5
Ma’an	3	0.5
Balqa	51	8.8
Jerash	3	0.5
Tafila	0	0.0
Madaba1	14	2.4
**Occupational field**		
Medical	138	23.8
Non-Medical	287	49.6
Unemployed	154	26.6
**Family income:( JD)**		
Less than 500	167	28.8
500–1000	289	50
More than1000	123	21.2
**Do you have a family member who has been diagnosed with cancer?**		
No	376	64.9
Yes	203	35.1

### Participants' knowledge regarding the prevalent and non-prevalent *cancer* types in Jordan and general causes of *cancer*

**Fig. 1:**
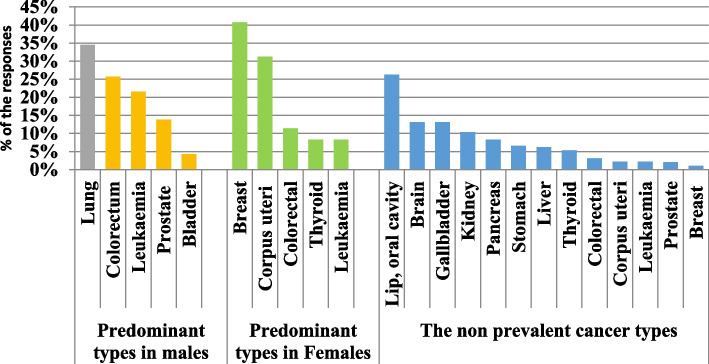
Knowledge of the participants regarding the prevalent and non-prevalent cancer types in Jordan

**Fig. 2:**
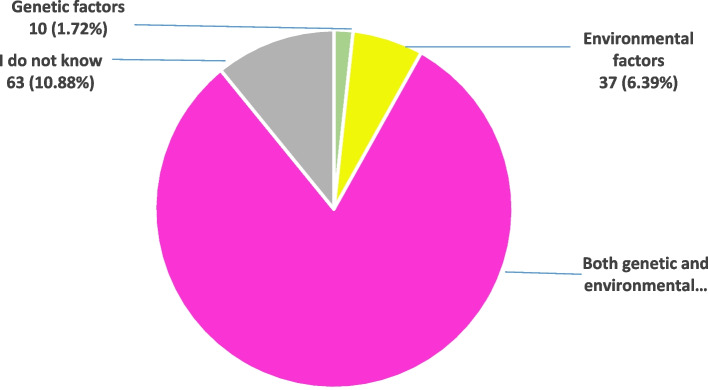
General causes of cancer identified by the study population

According to the GLOBOCAN estimates for 2020 in Jordan, the top 5 most frequent cancers in females are: Breast, Colorectal, Thyroid, Corpus uteri, and Leukaemia. In males, Lung, Colorectal, Bladder, Prostate, and Leukaemia are the top 5 most frequent cancers. When the participants were questioned regarding the most prevalent type of cancer in males, 34.54% identified lung and 25.73% identified colorectal cancer. Leukemia, prostate, and bladder cancers were least acknowledged as prevalent malignancies in males with only 21.59%, 13.82%, and 4.32% identifying them, respectively. When participants were questioned about the most prevalent type of cancer in females, 40.76% identified breast and 31.26% identified Corpus uteri while colorectal, thyroid, and Leukaemia were least acknowledged as common malignancies in women with only 11.40%, 8.29%, and 8.29% identifying them, respectively. According to GLOBOCAN estimates for 2020 in Jordan, Gallbladder, lip, and oral cavity are ranked 25 and 21 regarding prevalence by cancer site. Only 13.13% and 26.25% of this study samples recognized Gallbladder, and lip and oral cavity among the non-prevalent cancer types respectively (Fig. 1). Of the participants, 81% accurately recognized that environmental and genetic variables can contribute to cancer development (Fig. 2).

### General knowledge about environmental carcinogens and risk of *cancer* among the study population

Our study population was asked about several environmental carcinogens and cancer risk factors. Selected substances, agents, or exposures are listed in the NTP's 15th Report on Carcinogens as known human carcinogens.

Cancer-causing exposures, such as tobacco smoke, second-hand smoke, ionizing radiation, and UV radiation (sunlight) were highly recognized as cancer risk factors by 95.34%, 77.20%, 73.06%, and 57.86%, respectively. The majority (82.04%) of the participants identified alcohol as one of the cancer risk factors. However, respondents had a poor level of awareness related to cancer-causing substances such as wood dust, Nitrosamines, Aflatoxins, Formaldehyde, Naphthalene, Asbestos, Benzene, and Arsenic which were identified by only 12.44%, 26.08%, 27.81%, 32.82%, 33.85%, 35.06%, 35.23%, and 48.88%, respectively. About 55% of the participants could correctly identify coke oven emissions as one of the cancer risk factors.

Disappointingly, a significant portion of participants did not classify infectious pathogens such as the Epstein-Barr virus (EBV) (29.53%), Helicobacter pylori (H. pylori) (36.62%), HBV and HCV (36.79%), Human Papillomaviruses (HPVs) (49.74%) as factors that can cause cancer or increase the risk for cancer, but more than half of our sample (54.58%) recognized Human Immunodeficiency Virus (HIV) as a cancer risk factor.

A total of 75.13% of the participants believed that cancer does not necessarily directly arise as a result of exposure to a known carcinogen and 85.15% of the participants identified other factors that influence whether a person exposed to a carcinogen will develop cancer such as the amount of exposure, exposure duration and the individual’s genetic background (Table 2).

**Table 2:** Study population’s general knowledge about environmental carcinogens and cancer risk (Selected substances are listed in the Fifteenth Report on Carcinogens)

(*N* = 579)			
**1-Do you think the following is a risk factor for cancer? n (%)**			
	**No**	**Yes**	**Do not know**
Ionizing radiation (Radon and X-ray)	52 (8.98)	423 (73.06)	104 (17.96)
UV radiation (Sunlight)	171 (29.53)	335 (57.86)	73 (12.61)
Tobacco smoking	19 (3.28)	552 (95.34)	8 (1.38)
Secondhand smoke (people who are regularly around environmental tobacco smoke)	76 (13.13)	447 (77.20)	56 (9.67)
Drinking alcohol	48 (8.29)	475 (82.04)	56 (9.67)
**Cancer-Causing Substances**			
Aflatoxins	20 (3.45)	161 (27.81)	398 (68.74)
Arsenic	34 (5.87)	283 (48.88)	262 (45.25)
Asbestos	28 (4.84)	203 (35.06)	348 (60.1)
Benzene	121(20.9)	204 (35.23)	254 (43.87)
Naphthalene	70 (12.01)	196 (33.85)	313 (54.14)
Coke oven emissions	77 (13.3)	319 (55.09)	183 (31.61)
Formaldehyde	49 (8.46)	190 (32.82)	340 (58.72)
Wood dust	269 (46.46)	72 (12.44)	238 (41.10)
Nitrosamines	45 (7.77)	151 (26.08)	383 (66.15)
**Infectious Agents**			
Epstein-Barr Virus (EBV)	63 (10.88)	171 (29.53)	345 (59.59)
HBV and HCV	120 (20.73)	213 (36.79)	246 (42.48)
Human Immunodeficiency Virus (HIV)	92 (15.89)	316 (54.58)	171 (29.53)
Human Papillomaviruses (HPVs)	58 (10.02)	288 (49.74)	233 (40.24)
Helicobacter pylori (H. pylori)	133 (22.97)	212 (36.62)	234 (40.41)
**2-Does any exposure to a known carcinogen always result in cancer? n (%)**			
	435 (75.13)	69 (11.92)	75 (12.95)
**3-What are the factors that influence whether a person exposed to a carcinogen will develop cancer? n (%)**			
Amount of exposure	19 (3.28)		
Duration of exposure	28 (4.83)		
Individual’s genetic background	39 (6.74)		
All of the above	493 (85.15)		

### Information source regarding carcinogens and interest in learning about substances that are known to cause *cancer* in humans

Of the participants, 56.65% chose the internet as their first choice when looking for information about carcinogens and about 19% chose social media as their preferred source for knowledge, while a small percentage chose either healthcare providers (8.98%), books (6.91%), friends/relatives (4%), family/parents (3.80%) or radio (0.49%). Most of the study participants (82.38%) expressed interest in following the published lists of substances that are either known or reasonably expected to be human carcinogens based on the body of available scientific information. Upon questioning the participants about the best way to increase knowledge about cancer among Jordanians, 71.16% preferred the media, followed by campaigns (24.35%) and hospitals (4.49%) (Table 3).

**Table 3:** Source of information about carcinogens and interest in education on agents known to be human carcinogens, Jordan, 2023

Variable (*N* = 579)	Frequency (n)	Percentage (%)
**The last time you sought carcinogens’ knowledge, what was your priority portal?**		
Family/Parents	22	3.80
Friends/Relatives	23	4
TV	0	0
Radio	3	0.49
Internet	328	56.65
Books	40	6.91
Healthcare providers	52	8.98
Social media	111	19.17
**Are you interested in following the published lists of substances that, based on the available scientific evidence, are known or are reasonably anticipated to be human carcinogens?**		
Yes	477	82.38
No	102	17.62
**Best way in your opinion to increase cancer knowledge among Jordanians**		
Media	412	71.16
Campaigns	141	24.35
Hospitals	26	4.49

### Knowledge of *cancer* prevention among study participants

Most of the participants (88.95%) believed that cancer could be preventable. Upon questioning the participants about the best ways to help in cancer prevention, 45.94% chose finding precancerous conditions early and 29.36% chose changes in diet and lifestyle. Only 10.90%, 9.33%, 2.07%, 1.73%, and 0.67% of the participants thought that putting strict regulations in workplaces to reduce exposures to known carcinogens, quitting smoking, having HPV and HBV vaccination, limiting alcohol drinking, and limiting sun exposure could help cancer prevention respectively (Table 4).

**Table 4:** The study population’s general knowledge about cancer prevention, Jordan, 2023

	**Frequency (n)**	**Percentage (%)**
**Could cancer be preventable?**		
Yes	515	88.95
No	64	11.05
**Best ways in your opinion to help prevent cancer**		
Finding precancerous conditions early	266	45.94
Changes in diet and lifestyle	170	29.36
Put strict regulations in workplaces to reduce exposure to known carcinogens	63	10.90
Quitting smoking	54	9.33
Having HPV and HBV vaccination	12	2.07
Limiting alcohol drinking	10	1.73
Limiting sun exposure	4	0.67

### Differences in environmental carcinogens knowledge scores according to sociodemographic characteristics and family history

Among respondents, (55.6%, *n* = 322) had a knowledge score ≥ 8 indicating a good knowledge level, while (44.4%, *n* = 257) had a knowledge score < 8 indicating poor knowledge. There were no significant differences in the environmental carcinogen knowledge score among individuals concerning: sex, region of residence, and family history of cancer (p > 0.05 for all) whereas environmental carcinogen knowledge score was significantly higher among single participants, those within a younger age group (18–27 years old), higher educational levels, higher income levels, and those working in the medical field (p < 0.05 for all) (Table 5).

**Table 5:** Differences in knowledge scores according to sociodemographic characteristics and family history

Variables (*N* = 579)	Risk factors knowledge score		Statistical test	*P* value
	**Median**	**IQR**		
**Sex**				
Male	8	5–14	3.44	0.55
Female	8	5–12		
**Age (years)**				
18–27	10	6–15	38.5	**p < 0.01**
28–37	8	5–11		
38–47	8	5–11		
> 47	6	4–9.75		
**Marital status**				
Single	9	6–14.7	15.5	***p*** < 0.01
Married	8	5–11		
Divorced	8	4.7–16.2		
Widowed	4	1–7.5		
**Education level**				
Below University	6	4–11	2.12	***p* = 0.01**
University and above	8	5–12		
**Region of residence**				
Amman	8	5–12		
Zarqa	8	5–13.5	14.2	0.163
Irbid	8	5–15		
Mafraq	6	3–10.7		
Karak	10	10–13.7		
Ajloun	13.5	13–14		
Aqaba	19	15–19		
Ma’an	12	9–15		
Balqa	8	4.5–12		
Jerash	7	6–7.5		
Madaba	8.5	6–11		
**Occupational field**				
Medical	12	9–16.2		
Non-Medical	7	4–10	82.3	**p < 0.01**
Unemployed	7	5–10		
**Family income (JD):**				
Less than 500	8	5–12		**p < 0.01**
500–1000	8	5–12	9.94	
More than1000	10	7–14		
**Family history**				
No	8	5–12	3.76	0.80
Yes	8	5–13		

Mann Whitney test was used for comparison between 2 groups; Kruskall Wallis test for comparison between more than 2 groups

### Associations with environmental carcinogen knowledge

In the multivariate logistic regression analysis, age, marital status, and employment field (either medical or non-medical) were found to be significant predictors of the environmental carcinogen knowledge score (*p* < 0.05) (Table 6).

**Table 6:** Multivariate logistic regression of knowledge score with socio-demographic variables

Knowledge score	Adjusted OR	95% C.I		*P* value
**Variable**				
**Age (years)**				
18–24^a^	-	-	-	**-**
25–35	4.66	2.40–9.01	2.40–9.01	***p*< 0.01*p*< 0.01*p*< 0.01**
36–45	2.838	1.59–5.05	1.59–5.05	
> 45	2.274	1.26–4.10	1.26–4.10	
**Marital status**				
Single	1.84	1.07 -3.16	1.07 -3.16	**0.026**
Married	1.71	0.42 -6.86	0.42 -6.86	0.445
Divorced	0.81	0.13 -4.88	0.13 -4.88	0.826
Widowed^a^	-	-	-	-
**Educational level**				
Below University^a^	-	-	-	**-**
University and above	1.084	.662	1.775	0.78
**Occupational field**				
Medical	0.23	0.130—0.40		**p < 0.01p < 0.01-**
Non-Medical	0.16	0.089- 0.29		
Unemployed^a^	-	-		
**Family income (JD):**				
Less than 500^a^	-	-		**-**
500–1000	0.58	0.34–1.01		0.05
More than1000	0.64	0.39–1.03		0.07

*C.I* confidence interval, *OR* odds ratio

(*p* < 0.05), (*p* ≤ 0.01) significant

^a^Reference category

## Discussion

A precancerous lesion usually develops into a malignant tumour over the course of several stages, during which normal cells can turn into tumour cells and cause cancer. These alterations are the consequence of the interplay between a person's genetic makeup and three types of external factors including physical carcinogens, chemical carcinogens and biological carcinogens [13, 14]. Using a cross-sectional research methodology, we evaluated Jordanians' awareness of some of the reported environmental carcinogens as well as their general knowledge about cancer.

In this study, 81% of the participants properly recognized that both environmental and genetic variables can contribute to cancer development. Indeed, for years, environmental variables connected to cancer have been investigated for their ability to cause chromosome damage and mis-segregation and to induce mutations of proto-oncogenes and tumor suppressor genes, with non-mutagenic carcinogens also being observed [15, 16]. In Lebanon, Kabalan et al. reported similar results; noting that approximately 86% of the respondents agreed that the cause of cancer is both genetic and environmental [8]. Our findings are also supported by the results of a pilot study conducted in the United Arab Emirates, which found that two-thirds of research participants were aware that both genetic and environmental variables can play a role in the development of cancer [7].

According to the GLOBOCAN estimates for 2020 in Jordan, the top 5 most frequent cancers in females (ranked by cases) are: Breast, Colorectal, Thyroid, Corpus uteri, and Leukaemia. In this study, we found that only 40.76% correctly identified breast as the most prevalent type of cancer in females. Despite the presence of two major programs for early detection and screening for breast cancer in Jordan- the Jordan Breast Cancer Program (JBCP) and the Jordan Private Sector Project for Women's Health (PSP)_, public awareness about the prevalence of breast cancer among Jordanian females still needs further improvements. Raising awareness regarding the prevalent cancer types will enhance the success of the JBCP and the PSP’s efforts in breast cancer screening and early detection.

Results of this study also showed that only 34.54% of the respondents correctly recognized that lung cancer is the most prevalent cancer type among Jordanian males. In November 2022, The King Hussein Cancer Foundation (KHCF) launched an awareness campaign under the slogan “your smoke hurts my lungs”. The campaign aims to spread awareness about lung cancer, its causes and symptoms, and prevention methods, in hopes that such campaigns would contribute to increasing public awareness about cancer. Our results highlight the significance of a follow-up study to assess the campaign's effectiveness.

Although colorectal cancer is the second most common cancer in Jordan in both sexes [9], unfortunately, the study population exhibited a lack of knowledge regarding its prevalence as only 11.40% of the respondents identified it as prevalent in females and 25.73% identified it as prevalent in males. These results are similar to those reported by Jadallah et al., where they found that only 25.6% were aware that colorectal cancer is the second most common cancer in Jordan [17].

Results of this study demonstrated that participants were able to correctly identify some environmental carcinogens such as tobacco smoke (95.34%), alcohol (82.04%), secondhand smoke (77.20%), ionizing radiation (73.06%), UV radiation (Sunlight) (57.86%) and coke oven emissions (55.09%). These findings are consistent with those reported by Kabalan et al., in Lebanon where more than half of their studied sample had recognized smoking, tobacco exposure, nuclear rays, UV radiation, and alcohol as cancer risk factors [8]. Our results were also in line with those demonstrated by Ahmed et al. in UAE; who found that 77.6% of the participants considered nuclear radiation to be a risk factor for cancer development, and tobacco smoking and alcohol intake were identified as directly associated with cancer by 87.3% and 70.5% of the study population, respectively [7].

It is interesting to note that the results of a cross-sectional study of 2,100 adult residents in England showed that a significant percentage of people were not aware of the connection between alcohol and cancer [18]. Another study conducted by Doyle et al., which included a representative sample of 7,498 Irish adults aged 15 + years, reported that there was a lack of knowledge on the connection between alcohol consumption and breast cancer, specifically the dangers of exceeding the suggested low-risk limit, with only 21% of the respondents properly recognizing the connection [19]. The conservative religious culture in several Middle Eastern countries that forbids alcohol encourages the notion that alcohol consumption is harmful which may explain the high awareness level of the alcohol-cancer link among our study sample.

On the other hand, low awareness was demonstrated among our study sample regarding cancer-causing substances such as wood dust, Nitrosamines, Aflatoxins, Formaldehyde, Naphthalene, Asbestos, Benzene, and Arsenic. Kabalan et al. also reported that less than 50% of their participants had recognized naphthalene, asbestos, arsenic, and wood dust as cancer risk factors [8]. In addition, these results supported what was previously reported by Ahmed et al.; where they demonstrated a low awareness level regarding wood dust, Aflatoxins, Naphthalene, and Asbestos [7].

Infectious pathogens are potent and modifiable causes of cancer [20]. Disappointingly, a significant portion of the participants did not correctly classify pathogenic agents such H. pylori, EBV, HBV, HCV, and HPV as factors that can cause cancer or increase the risk of cancer. These results were consistent with those reported by Ahmed et al. [7]; where infections were the least recognized cancer risk factor in their study sample. In a study conducted by Munishi et al. in Tanzania; knowledge of cancer risks associated with HPV and HBC was lower at 17% and 37%, respectively [21]. On the other hand, Yamagiwa et al. demonstrated that cancer-causing viral and bacterial infections were among the highest perceived factors in a cross-sectional survey conducted in Japan [22]. Raising awareness of infectious agents as a potential cause of cancer is important since many cancer types can be avoided by preventing these diseases through immunization or early detection and treatment.

It is important to understand that while a carcinogen is a cancer-causing substance, exposure doesn’t always cause cancer. It may increase a person’s risk depending on factors such as the duration of the exposure f and the person’s genetics [3]. Our study population displayed a high awareness level regarding this issue as the majority of our study population agreed that exposure to a known carcinogen does not always result in cancer development and identified other factors that could have an impact such as the amount of exposure, duration of exposure and the individual’s genetic background.

Although social media platforms are increasingly being used as an information source, only 19% of this study population chose it as a portal to be used when seeking knowledge about carcinogens. More than 56% of our study sample preferred to go online and use internet websites to search for information about carcinogens. These results align with those reported by Akkour et al.; where they demonstrated that the internet was the most used source for cancer knowledge (75.2%), while only 5% of their study sample used social media as a source for cancer knowledge [23]. The majority of our population was interested in following the published lists of substances that are recognized or reasonably expected to be human carcinogens and preferred the media as a source for enhancing their knowledge. These findings demonstrate how important the media is becoming for people seeking access to health information.

Awareness about the preventability of cancer significantly influences the adoption of preventive measures. Most participants thought it was possible to prevent cancer. The best strategies to aid in cancer prevention, according to our study sample, are early detection of precancerous diseases and dietary and lifestyle modifications. Notably, even though tobacco smoke was widely acknowledged as a cancer risk factor (95.34%), only 9.33% of participants thought that stopping smoking could help prevent cancer. This finding lends further credence to the participants' beliefs that exposure to known carcinogens does not always lead to the development of cancer and that other factors, such as a person's genetic background, may also play a role in the development of cancer.

Among respondents of this study, 55.6% had a knowledge score ≥ 8 indicating a good knowledge level. In Saudi Arabia, Akkour et al. reported similar results; noting that the relative knowledge about cancer risk factors among participants was good (≥ 50% positive answers) [23]. As expected, participants with higher educational levels and individuals with occupations in the medical sector had a significantly higher knowledge score, highlighting the necessity of expanding health awareness campaigns to reach a wider range of individuals. Akkour et al. also reported that a higher educational level was associated with a higher level of awareness [23]. In contrast, in the study conducted by Ahmed et al., there was no relationship between the cancer knowledge score and education level. However, they also reported that respondents with occupations in the medical field had higher knowledge scores than the remaining respondents [7].

Additionally, it is worth noting that among participants of this study, the environmental carcinogen knowledge score was significantly higher in single participants and in the younger age group (18–27 years old). The Internet holds great potential to support information gathering and decision-making surrounding health education and self-care. Older adults, however, underutilize the Internet for health information searches. They experience greater challenges accessing and using eHealth tools relative to younger adults [24–26] which could explain the higher knowledge score observed in this study among the younger age group. In addition, respondents with a higher income level had a significantly higher knowledge score. This result is supported by findings from previous studies that investigated the relationship between cancer awareness and socioeconomic position and reported that low-income individuals were more likely to be less aware of the risk factors for cancer [27, 28].

### Strengths and limitations of the study

To the best of our knowledge, no previous studies in Jordan evaluated the population’s general knowledge about environmental carcinogens and cancer risk. This study is subject to some limitations. First, the recruitment was performed through a convenience sampling method by publishing the survey online which affects the generalizability of the results. Second, although online surveys can reach a large sample of respondents, our sample size was relatively small. This survey should be conducted with a larger, more representative sample size. We tried to overcome the possibility of response duplications by managing the Google Form settings to allow each participant to fill out the form only once.

## Conclusion

Results of this study highlight the need to enhance knowledge about lesser-known cancer risk factors such as wood dust, Nitrosamines, Aflatoxins, Formaldehyde, Naphthalene, Asbestos, Benzene, and Arsenic; as well as pathogenic agents such as H. pylori, EBV, HBV, HCV and HPV. The findings of this study also emphasize the necessity of raising awareness about the prevalent cancer types in Jordan which could enhance the efficacy of various initiatives aimed at cancer screening and early detection. The environmental and occupational risk factors for cancer are modifiable, and exposure to them can be reduced through several strategies. One effective approach is to create and disseminate public health messages to persuade people to minimize or completely avoid exposure to environmental cancer hazards.

## Data Availability

All relevant raw data will be made freely available by the authors upon request.

## Abbreviations

NTP: The National Toxicology Program
IARC: The International Agency for Research on Cancer
EBV: Epstein-Barr virus
HBV: Hepatitis B Virus
HCV: Hepatitis C Virus
HIV: Human Immunodeficiency Virus
HPVs: Human Papillomaviruses
JBCP: Jordan Breast Cancer Program
PSP: Private Sector Project for Women's Health
KHCF: The King Hussein Cancer Foundation
